# Reviewer acknowledgement 2013

**DOI:** 10.1186/1472-6939-15-6

**Published:** 2014-01-31

**Authors:** Fernando Marques

**Affiliations:** 1 BioMed Central, 236 Gray's Inn Road, WC1X 8HB London, UK

## Abstract

**Contributing reviewers:**

The Editors of *BMC Medical Ethics* would like to thank all our reviewers who have contributed to the journal in Volume 13 (2013).

## 

Jens Aagaard-Hansen

Denmark

Adewale Adisa

Nigeria

Nasser Aghdami

Iran

Alessandra Alciati

Italy

Saad Alghanim

Saudi Arabia

Judy Allen

Australia

Redhwan Al-Naggar

Malaysia

Emma Angell

UK

Berna Arda

Turkey

Atsushi Asai

Japan

Peter John Aspinall

UK

Mark Aulisio

US

Alireza Bagheri

Iran

Geoff Ball

Canada

Joshua Balsters

Switzerland

Stephen Barrie

UK

Philip Bejon

Kenya

Hassan Bella

Saudi Arabia

Jocelyne Rachelle Benatar

New Zealand

Cecile Bensimon

Canada

Mia Bergenmar

Sweden

Ian Berle

UK

Timothy Bickmore

US

Seiji Bito

Japan

Isra Black

UK

Robyn Bluhm

US

Pierre-Henri Bréchat

France

Annelien L. Bredenoord

Netherlands

Tania Bubela

Canada

Philippe Calain

Switzerland

Krysia Canvin

UK

Emily Carrier

US

Andrew Carson-Stevens

UK

Bernie Carter

UK

Alessandro Cavalcanti

Brazil

Ruth Chadwick

UK

Lei-Shih Chen

US

Murat Civaner

Turkey

Ronald Cole-Turner

US

Daniel Cook

US

Lorraine Corfield

UK

Isabel Cornejo

Chile

Andrew Courtwright

US

John Coverdale

US

Gillian Crozier

Canada

Sarah Damery

UK

Gail Davey

UK

Inmaculada de Melo-Martin

US

Jantina De Vries

South Africa

Clare Delany

Australia

Alice Desclaux

Senegal

Kris Dierickx

Belgium

Ioannis Dimoliatis

Greece

Charles Douglas

Australia

Edward Dove

Canada

Omur Elcioglu

Turkey

Bernice Elger

Switzerland

Maged El-Setouhy

Saudi Arabia

Miran Epstein

UK

Angel Estella

Spain

Nir Eyal

US

Zaher Fanari

US

Walid Faraj

Lebanon

Thomas Feeley

US

Shmuel Fennig

Israel

Joan Finegan

Canada

Leonard Fleck

US

Charles Foster

UK

Leslie Francis

US

Ruth Freeman

UK

John Fromson

US

Paul Wenzel Geissler

Norway

Rob George

UK

Elaine Gibson

Canada

Aaron Goldenberg

US

Thomas Grisso

US

Aldiouma Guindo

Mali

Erik Gustavsson

Sweden

Susanne Haga

US

Kristina Haglund

Sweden

Muhammad Hammami

Saudi Arabia

Seetharaman Hariharan

Trinidad and Tobago

Hossein Hatami

Iran

Jason Haukoos

US

Philip Hebert

Canada

Elizabeth Heitman

US

Gert Helgesson

Sweden

Maggie Hendry

UK

Kristien Hens

Netherlands

Peter Herissone-Kelly

UK

Bjorn Hofmann

Norway

Matthew Hunt

Canada

Jane Luise Hutton

UK

Saima Iqbal

Pakistan

Jonathan Ives

UK

Piotr Iwanowski

Poland

Steven Joffe

US

Kjell Arne Johansson

Norway

Yann Joly

Canada

Ralf Jox

Germany

Marko Jukic

Croatia

Annemarie Jutel

New Zealand

Gernot Kaiser

Germany

Ros Kane

UK

Chris Kaposy

Canada

Nancy Kass

US

Scott Y Kim

US

Sharon Kling

South Africa

Eike-Henner Kluge

Canada

Peter Knapp

UK

Jonathan Koffman

UK

Baburao Koneru

US

Sheldon Krimsky

US

Wendela Leeuwenburgh-Pronk

Netherlands

Richard Lessells

South Africa

Stephen Levine

US

Alex Limkakeng

US

Robert Liston

Canada

Beate Littig

Austria

Jayne Lucke

Australia

Neil Lunt

UK

Darryl Macer

Thailand

Jihad Makhoul

Lebanon

Phillipa Malpas

New Zealand

Julien Mancini

France

Vicki Marsh

Kenya

Patricia Marshall

US

Brian C. Martinson

Afghanistan

Francis Masiye

Malawi

Zubin Master

US

Bojana Matejic

Serbia

Anna Mavroforou

Greece

Michelle Mello

US

Victoria Miller

US

Maurice B Mittelmark

Norway

Bert Molewijk

Netherlands

Sassy Molyneux

Kenya

Edward Monico

US

Elaine Morgan

US

Johanna Muckenhuber

Austria

Narifumi Nakaoka

Japan

Proscovia Bazanya Namujju

Finland

Peter A Newman

Canada

Herman Nys

Belgium

Elke Birgit Ochsmann

Germany

Kieran O'Doherty

Canada

Ian Olver

Australia

Simon Outram

Australia

Aasim Padela

US

Andrew Papanikitas

UK

Michael Parker

UK

Claire Penn

South Africa

Wim Pinxten

Belgium

Steven Pipe

US

Rano Mal Piryani

Nepal

Sabine Pompeia

Brazil

Corinna Porteri

Italy

Rouven Christian Porz

Switzerland

Kameshwar Prasad

India

Faisal Qureshi

US

Flavia Ramos

Brazil

Kasper Raus

Belgium

Christoph Rehmann-Sutter

Germany

Cary Reid

US

Kevin Reid

US

Peter Bart Reiner

Canada

Stella Reiter-Theil

Switzerland

Eduardo Rodriguez

Chile

Marc Roelands

Belgium

Wendy Rogers

Australia

Dario Sacchini

Italy

Helen Sammons

UK

Lars Sandman

Sweden

Pamela Sankar

US

Nadja Schroder

Brazil

Doris Schroeder

UK

Vera Scott

South Africa

Clive Seale

UK

Janet Seeley

Uganda

Mary Seeman

Canada

Kavita Shah Arora

US

Faissal Shaheen

Saudi Arabia

Mary Shariff

Canada

Gil Siegal

US

Roberta Siliquini

Italy

Sisira Siribaddana

Sri Lanka

Dominic Sisti

US

Anna Smajdor

UK

Sanju Sobnach

South Africa

Merle Spriggs

Australia

Florian Steger

Germany

David Studdert

Australia

Antonella Surbone

US

Antonella Surbone

Italy

Mia Svantesson

Sweden

Fredrik Svenaeus

Sweden

Elisabeth Svensson

Sweden

Emmanouil Symvoulakis

Greece

Jacinta Tan

UK

Godfrey Tangwa

Cameroon

Noritoshi Tanida

Japan

Urmila Thatte

India

Paul Tiffin

UK

Sandra Titus

US

Antony Tobin

Australia

William Toffler

US

Tsuen-Chiuan Tsai

Taiwan

Steven Van de Walle

Netherlands

Lieve Van den Block

Belgium

Evert van Leeuwen

Netherlands

Ghislaine van Thiel

Netherlands

Colleen Varcoe

Canada

Jimmie Vaught

US

Joseph Verheijde

US

Geetika Verma

Canada

Claudio Verusio

Italy

Bettina von Helversen

Switzerland

Elizabeth Wager

UK

David Waisel

US

Mayyada Wazaify

Jordan

Edgar Whitley

UK

Nicola Williams

UK

Mark Yarborough

US

Daniella Zipkin

US

## Pre-publication history

The pre-publication history for this paper can be accessed here:

http://www.biomedcentral.com/1472-6939/15/6/prepub

